# Single-Cell ICP-MS in Combination with Fluorescence-Activated Cell Sorting for Investigating the Effects of Nanotransported Cisplatin(IV) Prodrugs

**DOI:** 10.1021/acs.analchem.3c02506

**Published:** 2023-08-03

**Authors:** Lucia Gutierrez-Romero, Elisa Blanco-González, Maria Montes-Bayón

**Affiliations:** † Department of Physical and Analytical Chemistry, Faculty of Chemistry, University of Oviedo, C/Julián Clavería 8, 33006 Oviedo, Spain; ‡ Health Research Institute of the Principality of Asturias (ISPA), Avda. Hospital Universitario s/n, 33011 Oviedo, Spain

## Abstract

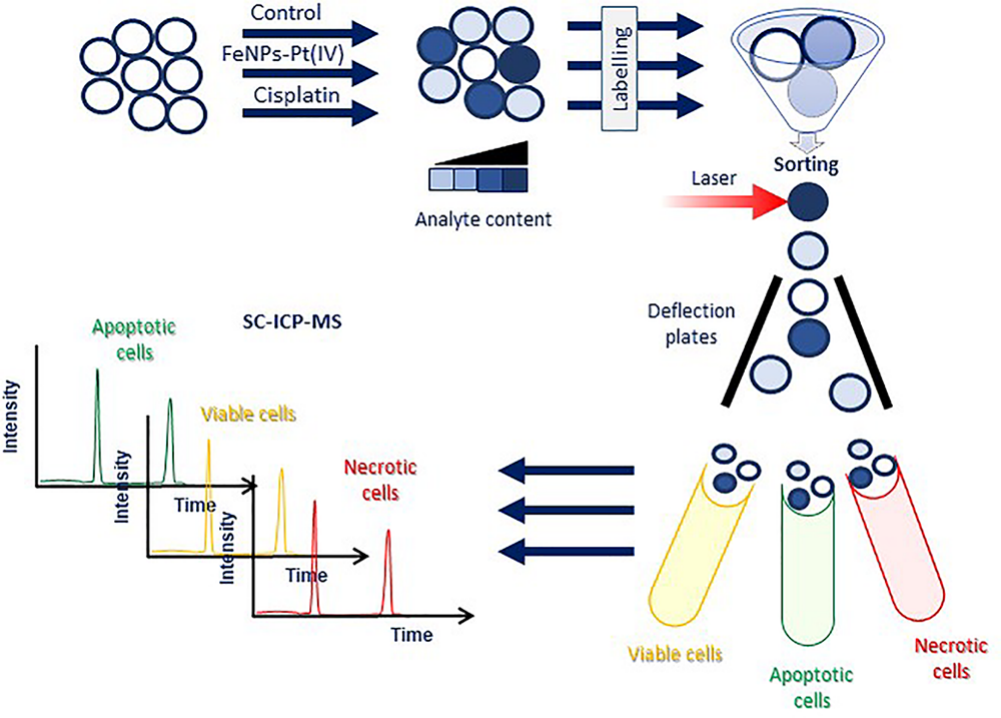

The combined use of fluorescence-activated cell sorting (FACS) and single-cell inductively coupled plasma mass spectrometry (SC-ICP-MS) is reported, for the first time, in this work. It is applied to evaluate the differences between the cellular uptake of ultrasmall iron oxide nanoparticles (FeNPs) loaded with cisplatin(IV) prodrug (FeNPs-Pt(IV)) and cisplatin regarding cell viability. For this aim, FACS is applied to separate viable, apoptotic, and necrotic A2780 ovarian cancer cells after exposing them to the nanotransported prodrug and cisplatin, respectively. The different sorted cell populations are individually analyzed using quantitative SC-ICP-MS to address the intracellular amount of Pt. The highest Pt intracellular content occurs in the apoptotic cell population (about 2.1 fg Pt/cell) with a narrow intercellular distribution when using FeNPs-Pt(IV) nanoprodrug and containing the largest number of cells (75% of the total). In the case of the cisplatin-treated cells, the highest Pt content (about 1.6 fg Pt/cell) could be determined in the viable sorted cell population. The combined methodology, never explored before, permits a more accurate picture of the effect of the intracellular drug content together with the cell death mechanisms associated with the free drug and the nanotransported prodrug, respectively, and opens the door to many possible single-cell experiments in sorted cell populations.

## Introduction

Nanodelivery systems, including liposomes or metallic nanoparticles, exhibit the capability to incorporate cisplatin (cis-diamminedichloroplatinum(II)) or its analogues (e.g., the cisplatin(IV) prodrug cis-diamminetetrachloroplatinum(IV)) into cells with enhanced efficiency.^1,2^ In the case of the Pt(IV) prodrugs, upon entrance into the cell cytosol, they undergo the chemical reduction to functional cisplatin(II) in a time-dependent manner resulting in a sustained release of the functional drug.^3,4^ In particular, ultrasmall iron oxide nanoparticles (<5 nm) coated with tartaric and adipic acids and loaded with cisplatin(IV) prodrug (FeNPs-Pt(IV)) have shown efficient incorporation into cells with the induction of apoptotic effects different from those observed for cisplatin.^5,6^ Therefore, this work aims to obtain deeper insights in the differential mechanisms of action of the two compounds (cisplatin and FeNPs-Pt(IV) nanoprodrug). For this aim, a direct correlation between intracellular Pt amounts in individual cells from previously sorted cell populations according to their apoptotic status will be conducted.

For cell sorting, fluorescence-activated cell sorting (FACS) is a special type of flow cytometry that allows the purification of individual cells based on the use of fluorescently tagged reagents, that recognize specific markers on the desired cell population.^7,8^ The cellular sorting among viable, apoptotic, and necrotic cells is usually conducted by FACS using different types of “viability dyes”.^9^ After initiating apoptosis, cells translocate phosphatidylserine (PS), an essential membrane phospholipid, from the intracellular-facing side of the cell membrane to the extracellular side of the membrane. Once on the cell surface, PS can be easily detected by fluorescently labeled annexin V, a protein that has a high affinity for PS. Another viability dye, propidium iodide (PI), cannot pass through the intact membrane of a live cell, but efficiently enters the cytoplasm and nuclei of dead cells where they intercalate noncovalently into DNA. Then, annexin V and propidium iodide (PI) dual staining in conjunction with a flow cytometer with sorting capacity and the appropriate software allows the purification of the individual cell populations (viable, apoptotic, and necrotic).

Here, we have combined the cell sorting capabilities of flow cytometry with the single-cell inductively coupled plasma mass spectrometry (SC-ICP-MS) experiments^10^ aimed to address the intracellular amount of Pt in individual cells of the previously sorted population.^11^ Although the ICP-MS elemental analysis of sorted cells has been shown before by using bulk determination,^12^ this is the first time to our knowledge that single-cell capabilities are demonstrated in combination with cell sorting. The intercellular variation in the uptake of specific metallodrugs can be essential to evaluate the initiation, for instance, of drug-resistance mechanisms.^13^ Therefore, here we propose a hyphenated tool to extract simultaneous biologically relevant information regarding the cell status with the cell-to-cell uptake variation of the drug.

## Experimental Section

### Materials

All solutions were prepared using 18 MΩ cm deionized water obtained from a PURELAB flex 3 (ELGA Veolia, Lane End, UK). Iron(III) chloride hexahydrate (98%, Sigma-Aldrich, Madrid, Spain) was used as the precursor for the nanoparticle synthesis. Sodium tartrate dihydrate (99–101%, Sigma-Aldrich) and adipic acid (99%, Sigma-Aldrich) were solubilized in 0.9% potassium chloride solution (Merck, Darmstadt, Germany) to be used as the nanoparticle coating agents. Ammonium acetate (>98%, Sigma-Aldrich) was used for the synthesis buffer and 5 mol·L^–1^ sodium hydroxide (Merck) was used for the nanoparticle precipitation.

RPMI 1640 Dulbecco’s culture medium, Tris-HCl buffered saline (TBS), and fetal bovine serum (FBS) were purchased from Gibco (Thermo-Fisher, Spain); plasmocin was obtained from InvivoGen (San Diego, USA); and trypsin was supplied by VWR-Avantor (Spain).

Annexin V-FITC Apoptosis Detection Kit was purchased form Sigma-Aldrich, and 30 000 and 3000 Da Pall Macrosep Advance centrifugal filter units were obtained from Pall Corporation (New York, USA).

### Instrumentation

All ICP-MS (inductively coupled plasma mass spectrometry) experiments for this work were performed using the triple quadrupole instrument iCAP TQ ICP-MS (Thermo Fisher Scientific, Bremen, Germany) working in the single-quadrupole (SQ)-none mode (no gas needed in the collision cell) for ^195^Pt^+^ monitoring. For single-cell analysis, the ICP-MS instrument was fitted with a high-performance concentric chamber from Glass Expansion (Glass Expansion, Australia). Cells were pumped into the system with a microflow syringe pump Chemyx Fusion 100-X Dual Syringe Infusion Only Pump (KR Analytical, Sandbach, United Kingdom) fitted with 1 mL Hamilton syringe (Nevada, USA) at 10 mL min^–1^. The recorded data were obtained in a time-resolved analysis mode during 2 min per analysis using a dwell time of 5 ms. Operational conditions are shown in Table S1.

For cell sorting experiments, BD FACSAriaTM IIu (BD Bioscience, Franklin Lakes, NJ, USA) from the Flow Cytometry and Cell Separation Unit of the Health Research Institute of the Principality of Asturias (ISPA) was employed for the separation of cells in different cell death degrees.

For centrifugation/ultrafiltration steps, a Biofuge Stratos Heraeus centrifuge (Thermo Fisher Scientific) was used.

### Cell Lines and Cell Culture

Two human ovarian cancer cell lines, A2780 and OVCAR-3, both sensitive to cisplatin in a higher (A2780) and lower (OVCAR-3) extension, were used in the experiments. The A2780 cell line was obtained from the Biotechnological and Biomedical Assays Unit at the Scientific and Technical Services (SCTs) of the University of Oviedo, and the OVCAR-3 cell line was purchased from the American Type Culture Collection (ATCC, Manassas, VA, USA).

Cell cultures were grown in 75 cm^2^ flasks at 37 °C in an atmosphere of 5% CO_2_ and a relative humidity of approximately 95% and maintained in RPMI 1640 medium supplemented with 10% FBS and 5 μg mL^–1^ of Plasmocin. Medium was replaced every 2–3 days after washing with TBS. After the desired treatments, cells were harvested by using trypsin in order to perform cell-sorting experiments.

### Synthesis of Cisplatin(IV) Prodrug Loaded Iron Oxide Nanoparticles

Ultrasmall iron oxide nanoparticles (FeNPs) coated with tartaric/adipic acids and loaded with cisplatin(IV) prodrug (FeNPs-Pt(IV)) were synthesized as presented in previous publications.^5,6^ Purification of the synthesized nanoparticles was performed by two ultrafiltration steps using first a 30 000 Da centrifugal filter and then a 3000 Da centrifugal filter.

For incorporation of the cisplatin(IV) prodrug, a solution of 5 mmol L^–1^ of the prodrug was incubated with the FeNPs during 6 h at room temperature under agitation. The excess of the prodrug was eliminated by ultracentrifugation using 3000 Da centrifugal filters. Characterization of the cisplatin(IV) prodrug-loaded iron oxide nanoparticles was carried out using developed strategies.^5^

### Cell Sorting

**Figure 1:**
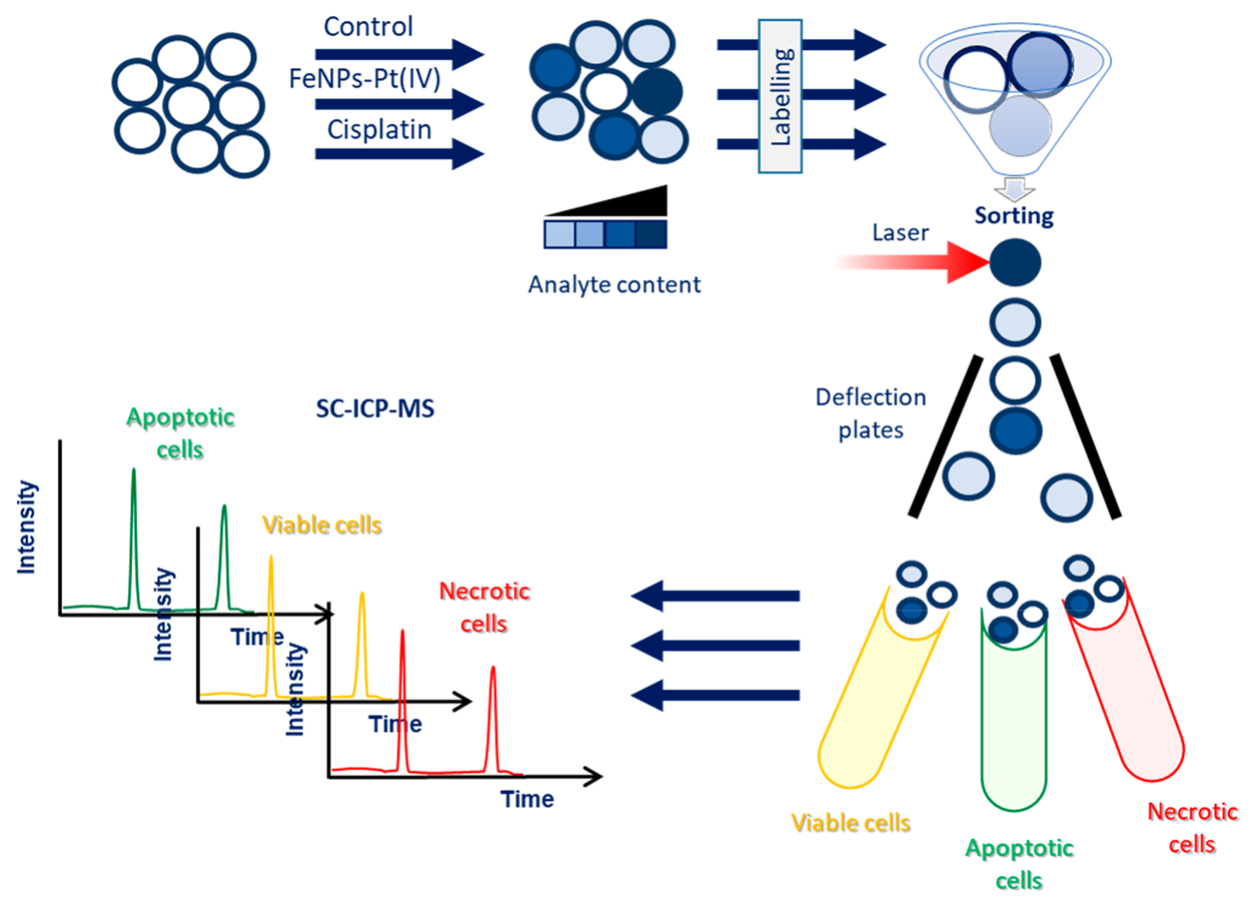
General scheme of the applied strategy of fluorescence-activated cell sorting combined to single-cell ICP-MS assay.

Cell apoptosis was measured following the Annexin V-FITC Apoptosis Detection Kit (Sigma-Aldrich) protocol with a few small changes. The kit uses annexin V conjugated with fluorescein isothiocyanate (FITC) to label phosphatidylserine (PS) on the membrane surface, and the kit includes propidium iodide (PI) to label the cellular DNA in necrotic cells. This combination allows the differentiation among early apoptotic cells (annexin V positive, PI negative), necrotic cells (annexin V positive, PI positive), and viable cells (annexin V negative, PI negative). Cells were grown in 75 cm^2^ flasks, where they were treated for 24 h with 40 μmol L^–1^ Pt in the form of FeNPs-Pt(IV) and cisplatin, respectively. Cells not exposed to the drugs served as control in each experiment. After the treatment, the medium was removed, and the cells were washed 3 times with TBS and harvested with trypsin. Cells were then precipitated by centrifugation to obtain a clean cell pellet. Two mL of the binding buffer supplied in the kit; 15 μL of annexin V -FITC conjugate and 3 μL of PI were added to the cells pellet for resuspension. After a 10 min incubation period at room temperature in the dark, cells were introduced into the cytometer for the cell counting and fluorescence-activated cell sorting. Annexin V-FITC is detected as a green fluorescence, and propidium iodide is detected as a red fluorescence. The sorted viable, apoptotic, and necrotic cells were individually collected in vials containing 300 μL of TBS and kept in ice until analysis by SC-ICP-MS. A total time of approximately 2 h passes from the sorting to the SC-ICP-MS analysis. From each cell status condition, a minimum number of 3 × 10^4^ cells were collected to obtain statistically representative results from SC-ICP-MS. The scheme of the conducted strategy is shown in Figure 1.

## Results and Discussion

### Global Cellular Uptake of the Drugs

**Figure 2:**
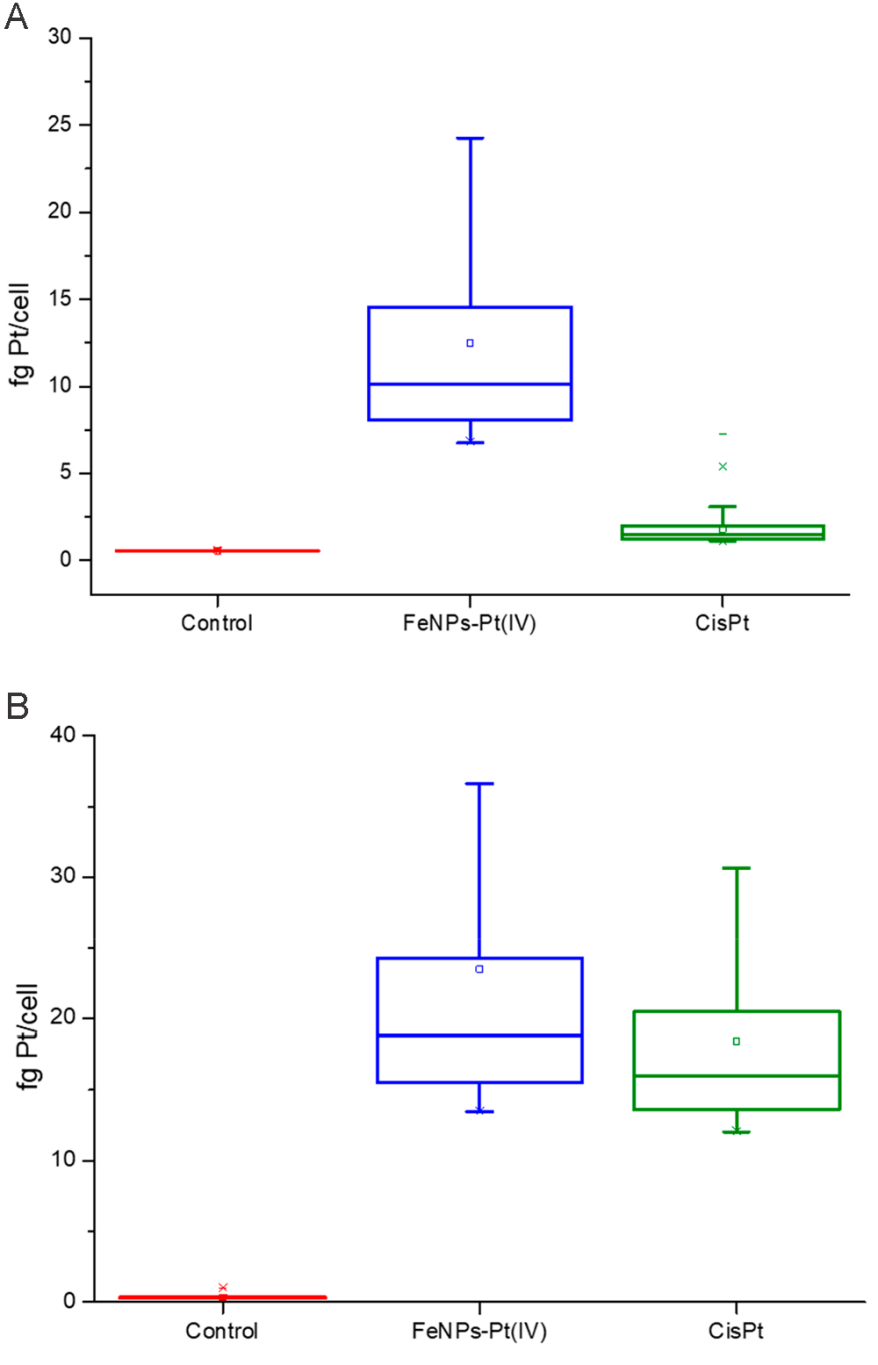
Intracellular amount of platinum (fg Pt/cell) in global cell populations of ovarian cancer lines (A) A2780 and (B) OVCAR-3. The box plot shows the results for control (red), FeNPs-Pt(IV) treated cells (blue), and cisplatin (green) treated cells.

The global cellular incorporation of the two platinum drugs was first studied using previously developed quantitative SC-ICP-MS strategies.^5,10^ It has to be pointed out that such high concentration of cisplatin is toxic for the A2780 cell model (LD_50_ about 12 μmol L^–1^ for 48 h of exposure)^14^ that shows higher sensitivity to cisplatin than the OVCAR-3 cell line.^15^ This was visually observed by the presence of some nonadherent A2780 cells after 24 h of cisplatin administration; these cells were discarded for further studies. On the other hand, no significant cell death was observed when they were treated with the FeNPs-Pt(IV) nanoprodrug at the same Pt concentration for 24 h. Figure 2a shows the intracellular amount of platinum in both treatments for A2780 cells that reaches levels of 10 fg Pt/cell in the case of the FeNPs-Pt(IV) and only about 2 fg Pt/cell in the case of cisplatin. Thus, the level of incorporation of Pt into living cells treated with the cisplatin(IV) prodrug loaded nanoparticles is significantly higher than in the case of treatment with free cisplatin. These differences, however, are cell-type-dependent.^16,17^ This was verified by repeating the same experiment using another ovarian cancer cell model (OVCAR-3) with different sensitivity for cisplatin. In this case, the results shown in Figure 2b reveal only slight incorporation differences between the cisplatin and the FeNPs-Pt(IV) nanoprodrug and substantially superior to those of A2780 cells. In this case, no signs of cell toxicity were observed in any of the treatments.

### Cell Sorting and SC-ICP-MS

For the coupling of cell sorting with the SC-ICP-MS, A2780 and OVCAR-3 cells were treated with the two platinum drugs as in the previous experiment. After washing cells were labeled with annexin V-FITC conjugate and then with PI according to the established protocol. These two labels do not require cell fixation or permeation of the cell membrane; thus, the losses of intracellular Pt should be negligible for this reason. Cells were then introduced into the flow cytometer, counted, and sorted according to the fluorescent labels into viable, apoptotic, and necrotic populations. The total cell number concentration obtained for each treatment and the sorted cell numbers are given in Table 1. The total cell number concentration of the cells exposed to cisplatin is approximately 70% of that obtained when using the FeNPs-Pt(IV) nanoprodrug in the A2780 and about 60% in the OVCAR-3, due to the cisplatin toxicity effects at this concentration previously described. The cell sorting results revealed also a different scenario regarding the distribution among viable, apoptotic and necrotic cells for cisplatin and the FeNPs-Pt(IV) nanoprodrug. In the case of cisplatin, approximately the same amount of viable (6 × 10^5^ cells) and apoptotic cells (7.6 × 10^5^ cells) is observed in both cell models. However, in the case of the exposure to the FeNPs-Pt(IV), about 75% of the total cells (1.5 × 10^6^ cells) are in the apoptotic status in the A2780 model. In the OVCAR-3, such balance could not be obtained due to the aggregation characteristics of this cell model that allowed that the sorted cells accounted only for 12% and 23% (in the case of cells treated with FeNPs-Pt(IV) and cisplatin, respectively) with respect to the total cell number concentration.

**Table 1:** Obtained Results for the Global and Sorted Cells Obtained after Exposure to 40 μmol L^–1^ Pt Concentration in the Form of Cisplatin or FeNPs-Pt(IV)

Cell model	Treatment	Total cell number concentration	Viable cells	Apoptotic cells	Necrotic cells
A2780	Cisplatin	1.4 × 10^6^	6 × 10^5^ (43%)	7.5 × 10^5^ (51%)	1.3 × 10^4^
OVCAR-3		2.7 × 10^6^	3 × 10^5^	3 × 10^5^	1.9 × 10^4^
A2780	FeNPs-Pt(IV)	2.0 × 10^6^	4.7 × 10^5^ (23%)	1.5 × 10^6^ (75%)	2.5 × 10^4^
OVCAR-3		4.8 × 10^6^	3 × 10^5^	2.1 × 10^5^	4.4 × 10^4^

**Figure 3:**
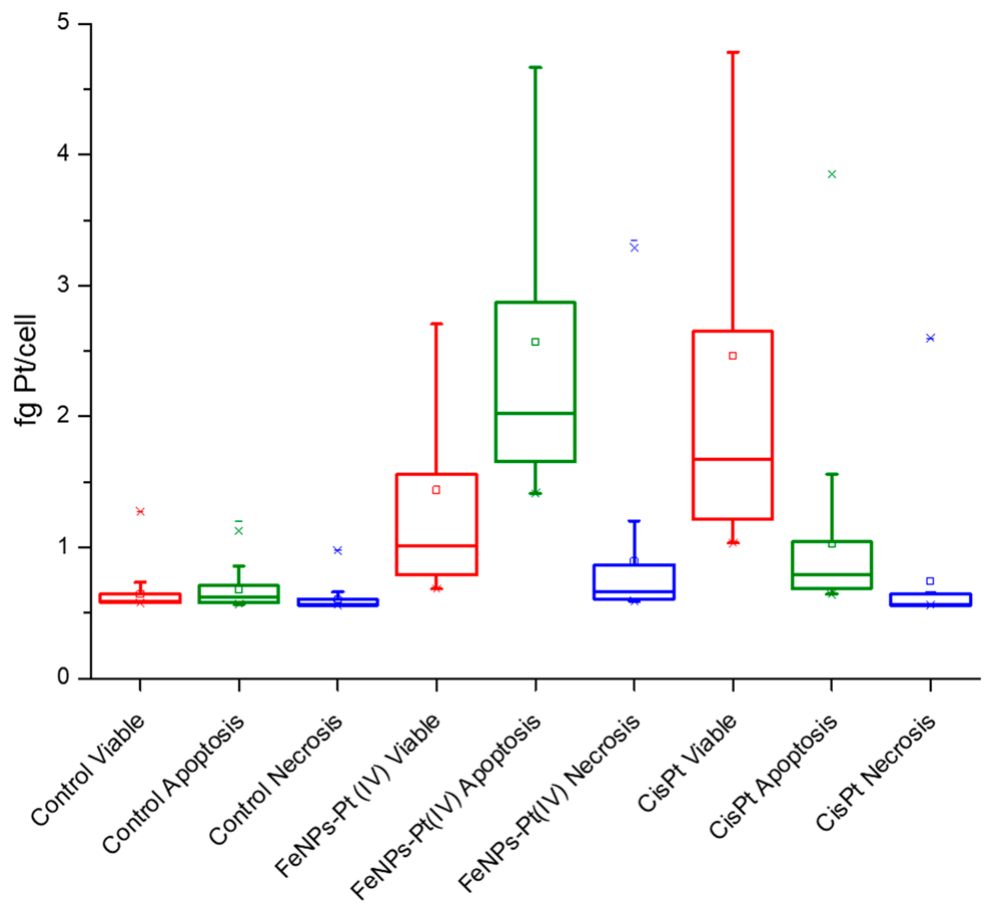
Intracellular amount of platinum (fg Pt/cell) in sorted cell populations of A2780 between viable (red), apoptotic (green), and necrotic (blue) for control, FeNPs-Pt(IV)-treated, and cisplatin-treated cells. The number of events considered on each treatment (excluding control) in the range of *n* = 60–1100.

Figure 3 shows the obtained results for the intracellular amount of Pt (fg Pt/cell) in the sorted cell populations for the control, cisplatin, and FeNPs-Pt(IV) nanoprodrug-treated A2780 cells. As can be seen, while in cisplatin-exposed cells the viable sorted population contains the highest intracellular Pt content, this occurs in the apoptotic sorted cells when treated with the FeNPs-Pt(IV). This could be explained as follows: cisplatin induced a significant cell death upon addition at this level of concentration (40 μmol L^–1^). Dead cells, probably containing the highest intracellular Pt content, were discarded once the pellet was collected for the sorting experiments. The remaining cells can cope with relatively high Pt doses without signs of toxicity and are viable, indicating a possible initiation of cell resistance mechanism. A similar trend has been also observed in the OVCAR-3 model (see Figure S1), but as seen in Figure 2, the differences among treatments are not as dramatic as in the A2780 model due to the higher resistance of these cells to the cisplatin. In any case, the highest intracellular Pt content is observed in the viable sorted cells exposed to cisplatin, while this occurs in the apoptotic sorted cells in the ones exposed to the FeNPs-Pt(IV) nanoprodrug.

On the other hand, the use of FeNPs-Pt(IV) nanoprodrug does not yield a significant cell death upon exposure. However, the cells showing the highest intracellular amount of Pt are the apoptotic sorted ones. Taking into account previous studies showing that the main mechanism of cell death when using this nanoprodrug is the apoptotic pathway, it can be foreseen that most cells in the A2780 model (1.5 × 10^6^ cells) will be defeated after some time by using the FeNPs-Pt(IV) nanoprodrug. In summary, in A2780 cells cisplatin produces a significant immediate cell death at 40 μmol L^–1^, but the surviving cells show lesser long-term signs of toxicity. On the other hand, the FeNPs-Pt(IV) nanoprodrug shows the slow release of the drug, yielding a prolonged apoptotic pathway over time. In other models, considered more resistant, like OVCAR-3, their use does not imply significant advantages with respect to the use of the free drug.

From a quantitative point of view, it is important to observe that for cisplatin-treated cells, the highest mass of Pt per cell corresponding to the sorted viable cells is similar to this measured in the complete cell population (see Figure 2). These results show a median of about 1.6 fg Pt/cell (with 75% of cells in the range of 1.2–2.6 fg Pt/cell) in the A2780 model. This value is significantly higher in the case of the OVCAR-3 where mean values in the cisplatin-treated sorted viable cells accounted for 5.1 fg Pt/cell (with 75% ranging 4–6 fg Pt/cell). However, in the case of using FeNPs-Pt(IV), the highest amount of intracellular Pt corresponds to the apoptotic cells and the mean is about 2.1 fg Pt/cell (range 1.65–3 fg Pt/cell) in the A2780 model. This result is significantly lower than the levels detected in the global cell population measurements (about 10 fg Pt/cell). We ascribe these differences to the possible presence of the FeNPs-Pt(IV) nanoprodrug on the cellular membrane when conducting the measurement of the global cell population, even after intense washing. During the labeling process, part of these species could be mobilized from the cell surface due to the reaction of one of the labeling reagents (annexin V-FITC conjugate) and be lost during sample treatment.

Intracellular platinum content of necrotic cells, on the other hand, was the lowest of all sorted populations. It is difficult to establish conclusions since, in this case, the cell membrane can be seriously compromised providing the leakage of Pt from the measured cells and contributing to a higher continuous background. As can be seen in Figure S2, there is a slight increase in the background ascribed to the possible Pt leakage previously described when compared the graphs of necrotic and viable cells. In any case, the measured event height takes the background contribution into account for the calculation of the Pt mass per cell. Furthermore, the number of necrotic sorted cells is between 1 and 1.5 orders of magnitude lower than the other populations; therefore, their contribution is not very significant.

## Conclusions

The coupling of cell sorting and single-cell ICP-MS has permitted the evaluation of the different toxicity mechanism when using nanocarriers of cisplatin(IV) prodrugs with respect to free cisplatin. Such a hybrid system allows correlating the intracellular amount of Pt on each individual sorted population but also addresses intracellular variation regarding such Pt content. In this case, the use of FeNPs-Pt(IV) shows higher incorporation into the cell cytosol in some cell models (A2780) and lower immediate systemic toxicity with respect to cisplatin. However, the controlled release of the drug from the nanoparticles yields a prolonged apoptotic pathway over time that is more the desired pathway when using nanocarriers. Therefore, this work also points out that the use of nanoparticles as carriers might represent an advantage depending on the cell type, and this has to be well-established beforehand. The proposed combined strategy opens the door to many future studies by combining the different sorting possibilities (e.g., cell cycle, cell phenotyping in complex cellular mixtures, etc.) with the elemental (or multielemental capabilities if ICP-TOF is applied) of SC-ICP-MS.

## Supporting Information Available

Table S1, instrumental conditions for SC-ICP-MS; Figure S1, SC-ICP-MS results obtained for the OVCAR-3 sorted cells; Figure S2, single-cell diagrams and their corresponding histograms for A2780 viable sorted cells treated with FeNPs-Pt(IV) and cisplatin treatment and A2780 necrotic sorted cells treated with FeNPs-Pt(IV) (PDF)
