# Supplementary material for: Single-Cell ICP-MS in Combination with Fluorescence-Activated Cell Sorting for Investigating the Effects of Nanotransported Cisplatin(IV) Prodrugs
Source: Anal Chem. 2023 Aug 3;95(32):11874–8. doi: 10.1021/acs.analchem.3c02506 (PMC10862375; doi:10.1021/acs.analchem.3c02506)
Supplement: Supplementary file 1 — ac3c02506_si_001.pdf [file ac3c02506_si_001.pdf]

Supporting Information

SINGLE CELL ICP-MS IN COMBINATION WITH FLUORESCENCE ACTIVATED CELL SORTING FOR INVESTIGATING THE EFFECTS OF NANOTRANSPORTED CISPLATIN (IV) PRODRUGS.

Lucia Gutierrez-Romero,[1,2] Elisa Blanco-González, [1,2] and Maria Montes-Bayón[1,2*]

[1]Department of Physical and Analytical Chemistry. Faculty of Chemistry. University of Oviedo. C/ Julián Clavería 8, 33006, Oviedo, Spain.

[2] Health Research Institute of the Principality of Asturias (ISPA). Avda. Hospital Universitario s/n, 33011, Oviedo, Spain.

*montesmaria@uniovi.es

The combined used of fluorescence activated cell sorting (FACS) and single cell inductively coupled plasma mass spectrometry (SC-ICP-MS) is reported, for the first time, in this work. It is applied to evaluate the differences between the cellular uptake of ultrasmall iron oxide nanoparticles (FeNPs) loaded with cisplatin (IV) prodrug (FeNPs-Pt(IV)) and cisplatin regarding cell viability.

TABLE OF CONTENTS

Table S1. Instrumental conditions for SC-ICP-MS

Figure S1. SC-ICP-MS results obtained for the OVCAR-3 sorted cells.

Figure S2. Single-Cell diagrams and their corresponding histograms for A2780 viable sorted cells treated with A) FeNPs-Pt(IV and B) cisplatin treatment and C) A2780 necrotic sorted cells treated with FeNPs-Pt(IV).

Table S1. Measurement parameters for SC-ICP-MS used to obtain the quantitative data.

Parameter	Value
Monitored Isotope	^{195}Pt
Measurement mode	SQ-none
Sample flow (µL/min)	10
Sheath Flow (Ar) (L/min)	0.638
Nebulizer Flow (Ar)(L/min)	0.5333
Dwell time (s)	0.005
Threshold value for event detection	4σ
Measuring time (s)	120
Pt calibration curve (ppb)	0-50
Transport efficiency of inorganic standards (measured with gold NPs)	78.50%

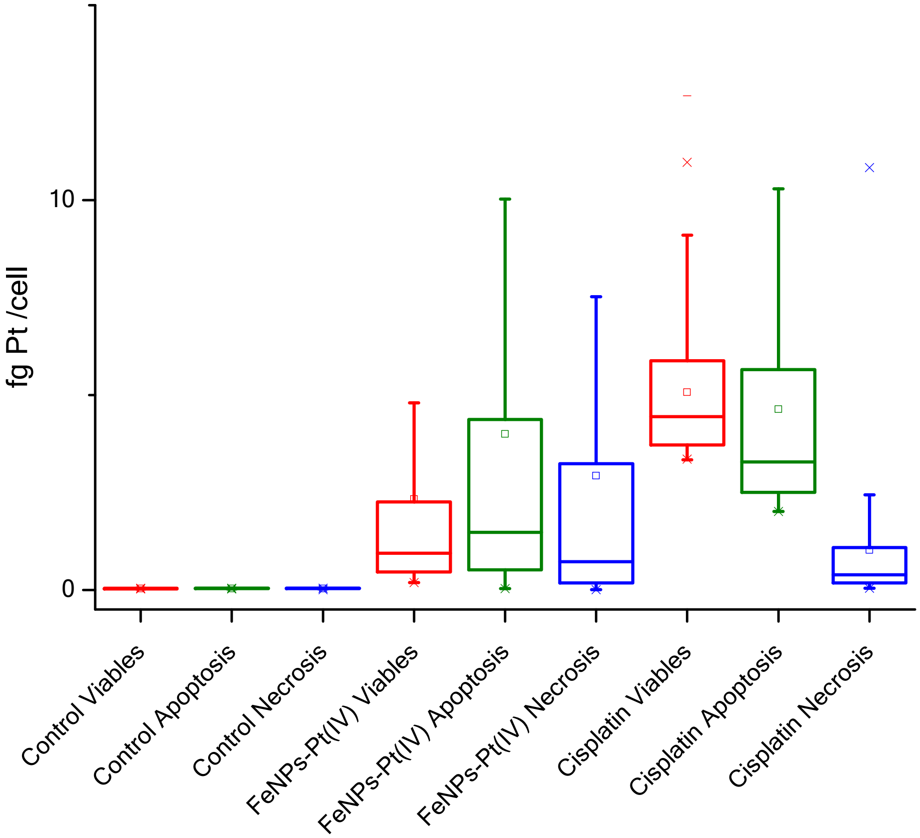

Figure S1. OVCAR-3 sorted cells and further measured by SC-ICP-MS.

A)

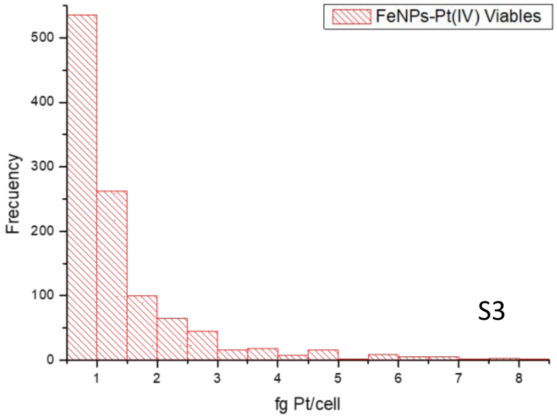

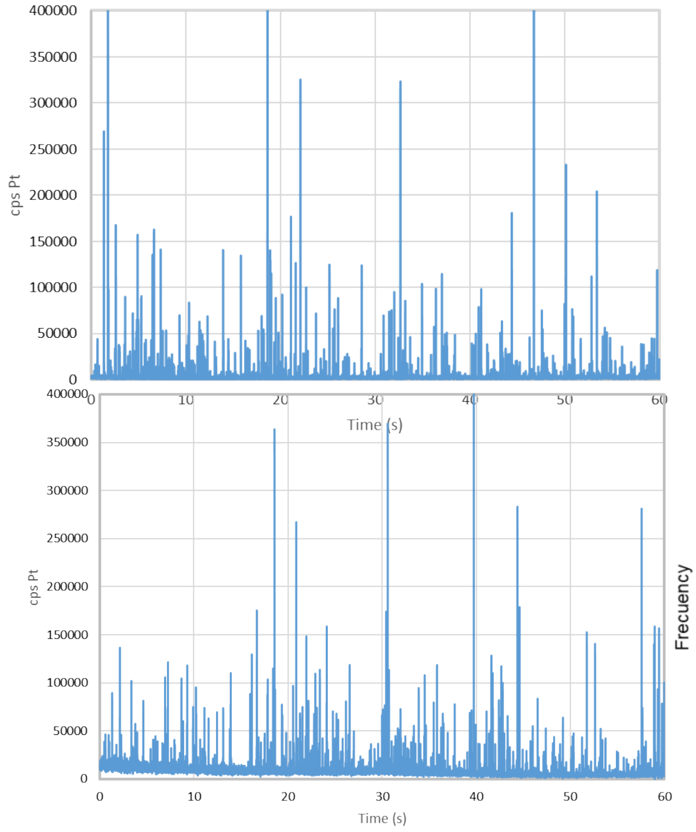
cps Pt
Time (s)

B)

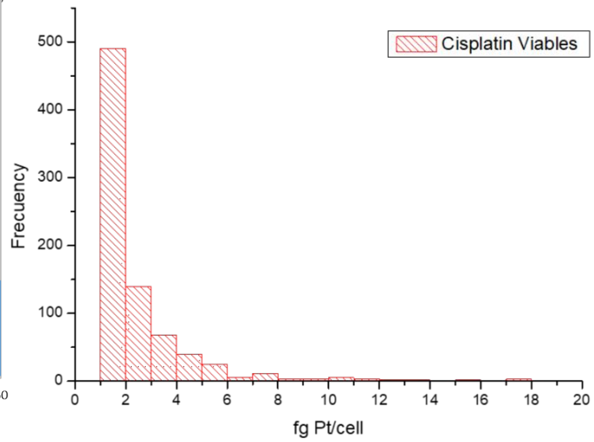
Cisplatin Viables
Frecuency
fg Pt/cell

C)

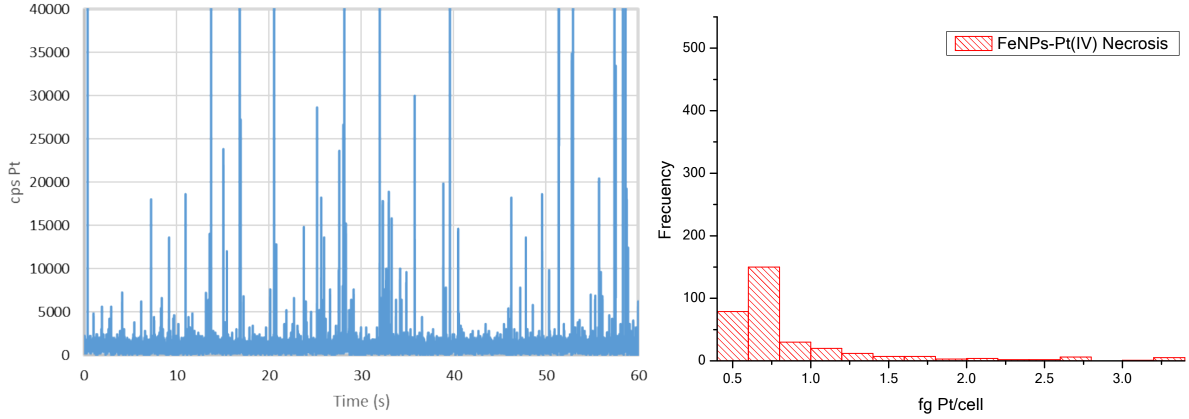
cps Pt
Time (s)
FeNPs-Pt(IV) Necrosis
Frecuency
fg Pt/cell

Figure S2. Single Cell Diagram and corresponding histograms of A2780 cells sorted viable and exposed to FeNPs-Pt(IV) (A) and cisplatin (B) respectively. Panel (C) corresponds to the necrotic sorted cells exposed to FeNPs-Pt(IV) (Y-axes magnified).
